# Breast cancer diagnosis using the fast learning network algorithm

**DOI:** 10.3389/fonc.2023.1150840

**Published:** 2023-04-27

**Authors:** Musatafa Abbas Abbood Albadr, Masri Ayob, Sabrina Tiun, Fahad Taha AL-Dhief, Anas Arram, Sura Khalaf

**Affiliations:** ^1^ Center for Artificial Intelligence Technology (CAIT), Faculty of Information Science and Technology, Universiti Kebangsaan Malaysia, Bangi, Selangor, Malaysia; ^2^ Department of Communication Engineering, School of Electrical Engineering, Universiti Teknologi Malaysia, (UTM), Johor Bahru, Johor, Malaysia; ^3^ Department of Computer Science, Birzeit University, Birzeit, Palestine; ^4^ Department of Communication Technology Engineering, College of Information Technology, Imam Ja’afer Al-Sadiq University, Baghdad, Iraq

**Keywords:** breast cancer, machine learning algorithms, data mining algorithms, fast learning network, Wisconsin breast cancer database, Wisconsin Diagnostic Breast Cancer

## Abstract

The use of machine learning (ML) and data mining algorithms in the diagnosis of breast cancer (BC) has recently received a lot of attention. The majority of these efforts, however, still require improvement since either they were not statistically evaluated or they were evaluated using insufficient assessment metrics, or both. One of the most recent and effective ML algorithms, fast learning network (FLN), may be seen as a reputable and efficient approach for classifying data; however, it has not been applied to the problem of BC diagnosis. Therefore, this study proposes the FLN algorithm in order to improve the accuracy of the BC diagnosis. The FLN algorithm has the capability to a) eliminate overfitting, b) solve the issues of both binary and multiclass classification, and c) perform like a kernel-based support vector machine with a structure of the neural network. In this study, two BC databases (Wisconsin Breast Cancer Database (WBCD) and Wisconsin Diagnostic Breast Cancer (WDBC)) were used to assess the performance of the FLN algorithm. The results of the experiment demonstrated the great performance of the suggested FLN method, which achieved an average of accuracy 98.37%, precision 95.94%, recall 99.40%, F-measure 97.64%, G-mean 97.65%, MCC 96.44%, and specificity 97.85% using the WBCD, as well as achieved an average of accuracy 96.88%, precision 94.84%, recall 96.81%, F-measure 95.80%, G-mean 95.81%, MCC 93.35%, and specificity 96.96% using the WDBC database. This suggests that the FLN algorithm is a reliable classifier for diagnosing BC and may be useful for resolving other application-related problems in the healthcare sector.

## 1 Introduction

**Figure 1:**
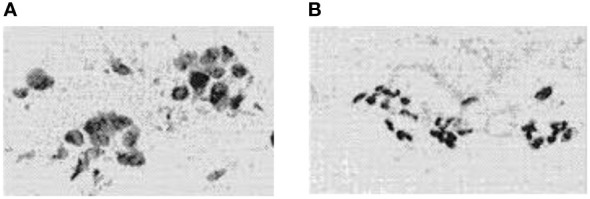
FNA’s digitized photos were: **(A)** is malignant and **(B)** is benign (4).

Uncontrolled cell growth within an organ leads to tumors, which can be cancer (1). Malignant and benign tumors are two different types of tumors. A cancerous or malignant tumor spreads and has an impact on human health and life. Although it is not spreading and does not pose a threat to life, the benign or non-cancerous tumor is not normal (2, 3). **Figure 1** illustrates digital representations of the FNA (fine needle aspirate) for both benign and malignant breast tumors. Malignant breast cancer is the expected result when growing cells are found in the breast tissue. One of the main causes of cancer death in women between the ages of 40 and 55 is breast cancer (BC) (5). In addition, BC is the second most common malignancy in the world after lung cancer (6). Early detection of BC will increase the chance of survival (7).

In recent decades, numerous fields, including emotion speech recognition (8), COVID-19 detection (9, 10), language identification (11–13), speaker gender identification (14), diabetic retinopathy detection (15), and voice pathology detection (16–18), have shown the effectiveness of data mining (DM) and machine learning (ML) techniques. As a result, significant attempts have recently been made to use DM and ML algorithms to diagnose BC (19, 20). These works, however, have a number of flaws, including the fact that the accuracy rates of the majority of the earlier works are still unsatisfactory and need improvement, that they have only been reviewed using one database, and that their performance has only been assessed using a limited number of assessment metrics without statistical analysis (21, 22).

The fast learning network (FLN) has recently become one of the best known ML algorithms (23). It is a double-parallel forward neural network (DPFNN), which is a parallel connection of a multilayer feedforward neural network (FNN) and a single-layer feedforward neural network (SLFN) (24, 25). The DPFNN’s output layer neurons also receive the external information directly through the input layer neurons, rather than just through the hidden layer neurons such as in the extreme learning machine and the standard neural network, which only receive external information after it has been modified (26). The input weights and hidden layer biases of the FLN are produced stochastically. Additionally, the values of the weights connecting the input layer with the output layer and the hidden layer with the output layer are analytically computed using least-square methods (27). In most situations, the FLN algorithm, which has fewer hidden neurons than other algorithms, can achieve good generalization performance with stability at a high speed (28).

Recently, the researchers prefer the FLN algorithm because it outperforms the conventional SVM (support vector machine) and BPNN (backpropagation neural network) (29, 30) particularly *via* the following: **a)** it eliminates overfitting, **b)** it has the ability to be implemented in both multi and binary classification tasks, and **c)** it has a similar capability to kernel-based SVM and works with a neural network structure. These components boost the FLN’s ability to produce exceptional learning outcomes. Nevertheless, as far as we are aware, no studies have used the FLN algorithm to detect BC. Furthermore, no studies have employed a variety of evaluation metrics and statistical analysis to assess the effectiveness based on the two separate databases Wisconsin Diagnostic Breast Cancer (WDBC) and Wisconsin Breast Cancer Database (WBCD). Consequently, the objectives of this work are as follows:

- To propose a new BC diagnosing classifier based on the FLN algorithm using two different databases WBCD and WDBC.
- To assess the proposed BC diagnosing classifier performance based on numerous assessment measurements such as G-mean, accuracy, specificity, F-measure, MCC (Matthews Correlation Coefficient), recall, ROC (receiver operating characteristic), precision, and execution time.
- To statistically assess the proposed BC diagnosing classifier performance based on mean, root mean square error (RMSE), and standard deviation (STD) in order to prove that the achieved results were not by chance.
- To evaluate the suggested BC classifier’s precision in contrast to the most recent works that utilized the same databases.

The rest of the paper is structured as follows: the related works are presented in Section 2. The materials and proposed technique are covered in Section 3. The findings of the experiments are explained in Section 4. Lastly, Section 5 provides the conclusion of the current study.

## 2 Related work

The research in (31) has proposed the ELM-ANN (extreme learning machine-artificial neural networks) approach for diagnosing the BC. The proposed ELM-ANN approach has been assessed based on the WBCD. The experimental results have shown that the proposed ELM-ANN approach outperformed its comparatives and achieved an accuracy that reached up to 96.40%.

Additionally, by utilizing the K-mean technique, the authors of (32) have presented a system for diagnosing the BC. Based on the BCWD, the suggested system’s evaluation was carried out. The results showed that the suggested method performed better than its counterparts and had an accuracy of 92.00%.

Further, the study in (33) has proposed six different classifiers LR (logistic regression), SVM, KNN (K-nearest neighbors), DT (decision tree), NB (naive Bayes), and RF (random forest) for BC diagnosing. These six different classifiers were assessed based on the WDBC database. The experiment results have revealed that the RF and SVM classifiers have achieved the highest performance with an accuracy that reached up to 96.50%.

In addition, the work in (34) has proposed a BC diagnosing classifier by using Kernel Neutrosophic c-Means Clustering as a feature weighting and random decision forest with Bayesian optimization as a classifier. The proposed system has been evaluated based on the WDBC database. The experimental outcomes have shown the superiority of the proposed system over its comparatives with an accuracy that reached up to 80.00%.

Furthermore, the research in (35) has tested five different ML algorithms, which are RF, SVM, KNN, DT, and LR in the diagnosis of the BC. The proposed five different ML algorithms have been assessed based on the WDBC database. The results have revealed that the SVM outperformed the other algorithms with an accuracy that reached up to 97.20%.

Additionally, the authors in (36) have proposed a hybrid BC diagnosing model by combining the genetic algorithm (GA) with the SVM for feature weighting and optimization of parameters. The proposed hybrid model was assessed based on the BCWD. The outcomes have demonstrated that the performance of the proposed model outperformed its comparatives with an achieved accuracy of 97.28%.

Also, the study in (37) has tested six different ML algorithms MLP (multilayer perceptron), GRU-SVM, NN (nearest neighbor), linear regression, SVM, and Softmax Regression (SR) in diagnosing the BC. The evaluation of the six different ML algorithms was conducted using the WDBC database. The experimental outcomes have revealed that among the six different ML algorithms, the MLP has achieved the highest performance with an accuracy rate that reached up to 99.04%.

Also, the work in (38) has proposed four different ML algorithms NB, SVM, DT, and KNN for BC diagnosing. The three different ML algorithms were assessed using the WBCD. The experimental results have shown that the SVM algorithm achieved the highest performance with an accuracy of 97.13%.

The research in (39) has proposed three different ML classifiers LR, SVM, and KNN for diagnosing the BC. The assessment of the three different classifiers was conducted based on the WBCD. The experiments outcomes have demonstrated that the KNN classifier has achieved the highest performance with an accuracy of 99.28%.

Moreover, the authors in (40) have proposed the averaged-perceptron machine-learning classifier for detecting the breast cancer. The proposed averaged-perceptron machine-learning classifier was evaluated based on the WBCD. The experiment outcomes have shown that the highest achieved accuracy of the proposed averaged-perceptron machine-learning classifier reached up to 98.40%.

In addition, the study in (41) has proposed a new breast cancer detection system by using the genetic programming with machine learning algorithms. The proposed system (i.e., genetic programming with machine learning algorithms) has been assessed based on the WDBC database. The experimental results have revealed that the highest performance of the proposed system (i.e., genetic programming with machine learning algorithms) was achieved with an accuracy of 98.24%.

The work in (42) has proposed numerous machine learning techniques such as SVM, RF, K-NN, DT, NB, LR, AdaBoost (AB), gradient boosting (GB), MLP, nearest cluster classifier (NCC), and voting classifier (LR+SVM) for diagnosing the breast cancer. All the proposed machine learning techniques were evaluated based on the WDBC database. The experiment results have demonstrated that the voting classifier (LR+SVM) achieved the highest performance with an accuracy reaching up to 98.77%.

The researchers in (43) have built an ensemble learning technique based on the Bayesian network and radial basis function (BN+RBF) for detecting the breast cancer. The assessment of the proposed ensemble learning technique (BN+RBF) was conducted using the WBCD. The experimental outcomes have shown that the highest performance of the proposed ensemble learning technique (BN+RBF) was accomplished with an accuracy of 97.42%. **Table 1** presents a summary of the prior works of the BC diagnosis using various ML and DM algorithms.

**Table 1:** Summary of the previous works.

Ref	Dataset	Method	Accuracy result	Disadvantage
(31)	BCWD	ELM-ANN	96.40%	• The accuracy result of the proposed studies still needs more improvement. • The proposed studies were assessed based on limited assessment measurements. • The evaluation of the proposed studies was conducted based on one database only. •The proposed studies did not conduct any statistical analysis in order to prove that the achieved results were not by chance.
(32)	BCWD	K-mean	92%	
(33)	WDBC	LR, SVM, KNN, DT, NB, and RF	96.50% with RF and SVM	
(34)	WDBC	KNCM + RDFBOA	80.00%	
(35)	WDBC	RF, SVM, KNN, DT, and LR	97.20% with SVM	• The proposed studies were assessed based on limited assessment measurements. • The evaluation of the proposed studies was conducted based on one database only. • The proposed studies did not conduct any statistical analysis in order to prove that the achieved results were not by chance.
(36)	BCWD	GA-SVM	97.28%	
(37)	WDBC	MLP, GRU-SVM, NN, linear regression, SVM, and SR	99.04% with MLP	
(38)	WBCD	NB, SVM, DT, and KNN	97.13% with SVM	
(39)	WBCD	LR, SVM and KNN	99.28% with KNN	
(40)	WBCD	Averaged-perceptron machine-learning classifier	98.40%	
(41)	WDBC	Genetic programming with machine learning algorithms	98.24%	
(42)	WDBC	SVM, RF, K-NN, DT, NB, LR, AB, GB, MLP, NCC, and voting classifier (LR+SVM)	98.77% with voting classifier (LR+SVM)	
(43)	WBCD	Ensemble learning technique based on (BN+RBF)	97.42%	

Based on all the abovementioned prior studies in diagnosing the BC, we can conclude the following:

- Most of the previous works such as (31–43) have been evaluated based on one database only.
- The accuracy results of most prior works, such as (31–34), are still not encouraging and need more enhancement.
- The performance of most former works, such as (31–43), were assessed utilizing a limited set of evaluation metrics (i.e., recall, accuracy, precision, specificity, and F-measure).
- The performance assessment of most previous works such as (31–43) have not conducted any statistical analysis in order to prove that the achieved results were not by chance.

## 3 Proposed method

**Figure 2:**
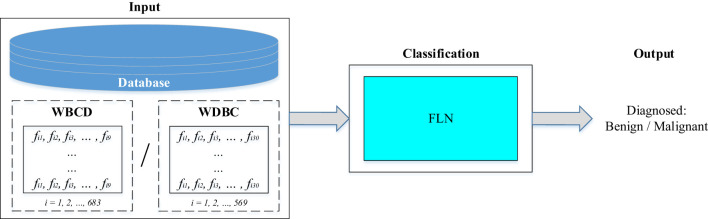
Diagram of the proposed breast cancer diagnosis classifier.

This work proposes a breast cancer diagnosis classifier based on the utilization of the FLN algorithm. Two different standard databases (i.e., WBCD with nine extracted features and WDBC with 30 extracted features) were used as input to evaluate the performance of the proposed FLN algorithm in diagnosing the breast cancer. The FLN algorithm in the classification stage is used to diagnose whether the input’s sample is benign or malignant. **Figure 2** depicts the proposed breast cancer diagnosis classifier diagram.

### 3.1 Database

In this work, two different standard databases (i.e., WBCD and WDBC) were used to evaluate the performance of the proposed FLN algorithm in diagnosing the breast cancer. The two different databases (i.e., WBCD and WDBC) have been provided in form of features. Deep descriptions and details of both databases and their extracted features are provided in the following:

• **WBCD:** The WBCD was obtained from the University of Wisconsin Hospital (44). There are 699 samples altogether in the WBCD. However, 16 samples in the WBCD had missing values. In this study, all 16 samples with missing values were eliminated, and only 683 samples were taken into consideration (i.e., 458 samples for the benign category and 241 samples for the malignant category). A deep description and explanation of the WBCD are provided in (45). All the experiments of the current work are applied based on dividing the database into 30% for testing purposes and 70% for training purposes. **Table 2** presents the WBCD which has been utilized in this work.

**Table 2:** Description of the WBCD which has been utilized in this work.

Category	Total number of samples	Number of training samples	Number of testing sample	Label
Malignant	239	167	72	1
Benign	444	311	133	2

The WBCD was provided in a form of features where it contains nine features along with the label of the class (i.e., benign category or malignant category) and the ID number of the subject. These nine features are clump thickness, uniformity of cell size, uniformity of cell shape, marginal adhesion, single epithelial cell size, bare nuclei, bland chromatin, normal nucleoli, and mitoses. The values of these features are integers in the range of (1–10) where the 10 value refers to the critical state. **Table 3** illustrates the nine features of the WBCD. More details on the WBCD features are provided in (45).

**Table 3:** Illustration of the WBCD features (45).

Feature number	Feature	Feature range
1	Clump thickness	1-10
2	Uniformity of cell size	1-10
3	Uniformity of cell shape	1-10
4	Marginal adhesion	1-10
5	Single epithelial cell size	1-10
6	Bare nuclei	1-10
7	Bland chromatin	1-10
8	Normal nucleoli	1-10
9	Mitoses	1-10

• **WDBC database:** The WDBC database has been downloaded from the UCI website, which is a machine-learning repository (46). The WDBC database consists of tumor features that are computed from a digital image of the FNA of the breast mass. The total number of samples in the WDBC database is 569 samples (i.e., 357 samples for the benign category and 212 samples for the malignant category). A deep description and explanation of the WDBC database are provided in (45). All the experiments of the present work are implemented based on dividing the database into 30% for testing purposes and 70% for training purposes. **Table 4** depicts the WDBC database which has been used in this work.

**Table 4:** Depiction of the WDBC database which has been used in this work.

Category	Total number of samples	Number of training samples	Number of testing samples	Label
Malignant	212	148	64	1
Benign	357	250	107	2

The WDBC database was provided in a form of 32 tumor features that have been calculated from a digital image of the breast mass FNA. These 32 tumor features represent **i)** the ID number of the subject, **ii)** the label of the class, which refers to whether the subject belongs to a benign category or malignant category, and **iii)** 30 actual tumor features. For each subject, 10 characteristics of the cell nuclei (visual in the digital image of the breast FNA) are obtained: these are texture, radius, area, perimeter, compactness, smoothness, symmetry, concavity, fractal dimension, and concave points.

where

Radius: mean of distances from the center to points on the perimeter;

Texture: standard deviation of gray-scale values;

Perimeter: size of the core tumor;

Area;

Smoothness: local variation in radius lengths;

Compactness: perimeter^2^/area—1.0;

Concavity: severity of concave portions of the contour;

Concave points: number of concave portions of the contour;

Symmetry; and

Fractal dimension: coastline approximation—1.

Then, different measurements such as standard error, mean, and maximum of these 10 characteristics are computed, which results in 30 features. **Table 5** depicts these measurements, which represents the tumor features in the WDBC database. More details on these features of the WDBC database are provided in (45).

**Table 5:** Description of the WDBC database features (45).

Feature number	Feature	Feature range		
		Mean	Standard error	Maximum
1	Radius	6.98–28.11	0.112–2.873	7.93–36.04
2	Texture	9.71–39.28	0.36–4.89	12.02–49.54
3	Perimeter	43.79–188.50	0.76–21.98	50.41–251.20
4	Area	143.50–2501.00	6.80–542.20	185.20–4254.00
5	Smoothness	0.053–0.163	0.002–0.031	0.071–0.223
6	Compactness	0.019–0.345	0.002–0.135	0.027–1.058
7	Concavity	0.000–0.427	0.000–0.396	0.000–1.252
8	Concave points	0.000–0.201	0.000–0.053	0.000–0.291
9	Symmetry	0.106–0.304	0.008–0.079	0.157–0.664
10	Fractal dimension	0.050–0.097	0.001–0.030	0.055–0.208

### 3.2 FLN algorithm

**Figure 3:**
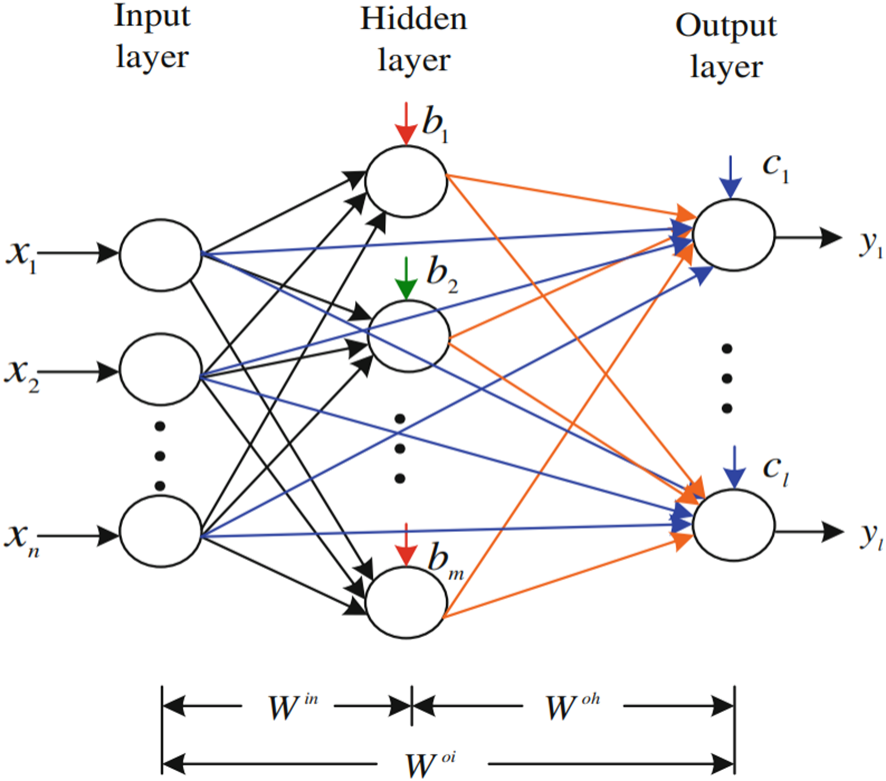
The FLN algorithm diagram.

FLN is a double-parallel forward artificial neural network proposed by (47). The FLN algorithm is based on the least-square techniques. **Figure 3** presents the general FLN algorithm diagram, and it is followed by a deep explanation of the FLN algorithm.

Assume that *N* refers to arbitrary distinct samples {*x_i_, y_i_*}, where *x_i_ = [x_i1_, x_i2_… x_in_]^T^* ∈ *R^n^* is a vector of the *i_th_* training sample with *n* dimension, and y_i_ = [*y_i1_, y_i2_ … y_il_*]^T^ ∈ *R^l^* is a vector of the *i_th_* target with the *l* dimension.

According to **Figure 3**, the FLN has *m* nodes in the hidden layer. **W** *^in^* refers to the matrix of the input weights with (*m × n*) dimension that links the input-layer nodes and hidden-layer nodes, while **b** = [*b_1_, b_2_ … b_m_*] ^T^ represents the matrix of the hidden-layer node biases. In addition, **W** *^oh^* denotes the weights’ matrix with (*l × m*) dimension that links the hidden-layer nodes with the output-layer nodes. **W** *^oi^* refers to the weights’ matrix with (*l × n*) dimension that links the input-layer nodes with the output-layer nodes. **c** = [*c_1_, c_2_ … c_l_*] ^T^ represents the matrix of the output-layer node biases. The active functions of the output-layer nodes and hidden-layer nodes are f(·) and g(·) , respectively. When the output-layer nodes’ biases **c** = [*c_1_, c_2_ … c_l_*] ^T^ are set equivalent to zeros, it will be ignored in the active function of the output-layer nodes. Consequently, the FLN algorithm mathematical model is depicted as follows:


(1) $$\{\begin{array}{l} y_{j1}=\sum_{r=1}^{n}W_{1r}^{oi}x_{jr}+c+\sum_{k=1}^{m}W_{1k}^{oh}g\left(b_{k}+\sum_{t=1}^{n}W_{kt}^{in}x_{jt}\right)\\ y_{j2}=\sum_{r=1}^{n}W_{2r}^{oi}x_{jr}+c+\sum_{k=1}^{m}W_{2k}^{oh}g\left(b_{k}+\sum_{t=1}^{n}W_{kt}^{in}x_{jt}\right),\;j=1,\;2,\cdots ,N\\ \vdots \\ y_{jl}=\sum_{r=1}^{n}W_{lr}^{oi}x_{jr}+c+\sum_{k=1}^{m}W_{lk}^{oh}g\left(b_{k}+\sum_{t=1}^{n}W_{kt}^{in}x_{jt}\right) \end{array}$$

Moreover, it could be represented as follows (see Equation 2):


(2) $$y_{j}=f\left(W^{oi}x_{j}+c+\sum_{k=1}^{m}W_{k}^{oh}g\left(W_{k}^{in}x_{j}+b_{k}\right)\right)\;,\;j=1,\;2,\cdots ,N$$

where:

$W^{oi}=\left[{\text{W}}_{1r}^{oi},{\text{W}}_{2r}^{oi}\ldots {\text{W}}_{lr}^{oi}\right]$ refers to the weight vector that is linking the *r_th_* input-layer node with the output-layer nodes. $W_{k}^{oh}=\;{\left[W_{1k}^{oh},W_{2k}^{oh}\ldots W_{lk}^{oh}\right]}^{T}$ represents the weight vector that is linking the *k_th_* hidden-layer node with the output-layer nodes. Also, $W_{k}^{in}={\left[W_{k1}^{in},W_{k2}^{in}\ldots W_{kn}^{in}\right]}^{T}$ denotes the weights vector that is linking the *k_th_* hidden-layer node and the input-layer nodes. The hidden-layer nodes’ output (**G**) are computed as the following equation:


(3) $$\begin{array}{c} G\left(W_{1}^{in},\cdots ,W_{m}^{in},b_{1},\cdots ,b_{m},x_{1},\cdots ,x_{N}\right)=\\ {\left[\begin{array}{ccc} g\left(W_{1}^{in}x_{1}+b_{1}\right) & \cdots & g\left(W_{1}^{in}x_{N}+b_{1}\right)\\ \vdots & \ddots & \vdots \\ g\left(W_{m}^{in}x_{1}+b_{m}\right) & \cdots & g\left(W_{m}^{in}x_{N}+b_{m}\right) \end{array}\right]}_{m\times N} \end{array}$$

The output weights’ matrix $\hat{W}=\left[{\text{W}}^{oi}{\text{W}}^{oh}\right]$ could be determined by the inverse of Moore–Penrose generalization (see Equation 4).


(4) $$\;\;\;\;\;\;\;\;\;\;\;\;\;\;\;\;\;\;\;\;\;\;\;\;\;\;\hat{W}=Y{\left[\begin{array}{c} X\\ G \end{array}\right]}^{+}=YH^{+}\;\;\;\;\text{where}\;H=\left[\begin{array}{c} X\\ G \end{array}\right]$$

$W^{oi}\text{ and }W^{oh}$ are computed as follows (see Equation 5):


(5) $$\;\;\;\;\;\;\;\;\;\;\;\;\;\;\;\;\;\;\;\;\;\;\;\;\;\;\;\;\;\;\;\;\;\;\;\;\begin{array}{c} W^{oi}=\hat{W}\left(1:l,1:n\right)\\ W^{oh}=\hat{W}\left(1:l,n+1:\left(n+m\right)\right) \end{array}$$

where l is the number of the output nodes (i.e., number of classes); n is the number of the input nodes (i.e., number of features); and m is the number of the hidden nodes.

Suppose that *N* is the given training set {*x_i_, y_i_*}, where *x_i_ = [x_i1_, x_i2_… x_in_]^T^* ∈ *R^n^* and y_i_ = [*y_i1_, y_i2_ … y_il_*]^T^ ∈ *R^l^*, activation function g(·) , and *m* is the hidden-layer nodes’ number, where

*x_i_* = the input, which is the extracted features (nine extracted features for the WBCD and 30 extracted features for the WDBC database).

*y_i_* = the true value (expected output).

Subsequently, the FLN algorithm learning procedure would be summarized as the following steps:

INPUT: training-set *N*

Step 1: Generate the **W** *^in^* and **b** (i.e., the input weights and biases) matrices randomly in the range of (–1, 1) for the input weights and [0, 1] for the biases.

Step 2: Compute the hidden-layer output matrix by using Equation (3).

Step 3: Calculate the combination matrix ( $\hat{W}$ ) by using Equation (4).

Step 4: Calculate the FLN algorithm parameter model by using Equation (5).

OUTPUT: the random generated **W** *^in^* and **b** (i.e., the input weights and biases) and the analytically computed **W** *^oi^* and **W** *^oh^* (i.e., weight values that connect the input layer with the output layer and the hidden layer with the output layer) by using the least-square method.

Once the learning process of the FLN algorithm is done, the obtained FLN model is tested on the testing data and its performance evaluated based on several evaluation measurements.

## 4 Experiments setup, results, and discussions

In this study, several experiments were implemented to assess the proposed FLN algorithm’s performance in diagnosing the BC using two databases WBCD and WDBC. These experiments were performed based on varying the hidden neuron number in the range of [25–200] with 25 increment steps. In order to statistically assess the effectiveness of the suggested FLN algorithm, a total of eight experiments were conducted, with each experiment being run 50 times. As in the literature (48, 49), the eight experiments were run based on dividing the database into 30% for testing purposes and 70% for training purposes. Additionally, all experiments were carried out using the MATLAB R2022a programming language on a computer that has Windows 10 Pro, 12 GB of RAM, and an Intel Core i7 running at 3.60 GHz. The outcomes of each run were assessed based on numerous evaluation measurements such as accuracy, precision, recall, F-measure, G-mean, MCC, and specificity. The mathematical calculation of these assessment measurements is depicted in Equations 6–12 (50, 51).


(6) $$\text{Recall}=\frac{TP}{TP\;+\;FN}$$

(7) $$\text{Accuracy}=\frac{TP\;+\;TN}{TP\;+\;TN\;+\;FP\;+\;FN}$$

$\text{F}-\text{measure}=\frac{2\;\times \;Precision\;\times \;Recall}{Recall\;+\;Precision}$ (8)


(9) $$\text{Precision}=\frac{TP}{TP\;+\;FP}$$

(10) $$\text{G}-\text{mean}=\sqrt{\text{Specificity}\times \text{Recall}}$$

(11) $$\text{Specificity}=\frac{TN}{TN\;+\;FP}$$

(12) $$\text{MCC}=\;\frac{\left(\text{TP}\times \text{ TN}-\text{FP}\times \text{ FN}\right)}{\sqrt{\left(TP+FP\right)\left(TP+FN\right)\left(TN+FP\right)\left(TN+FN\right)}}$$

where TP is true positive, TN is true negative, FP is false positive, and FN is false negative.

The 50 runs’ results of each experiment were used to calculate the mean, RMSE, and STD in order to statistically assess the proposed FLN algorithm’s performance in diagnosing the BC. These three assessments are considered the most common statistical evaluation measures (52, 53). The mean measures how close the overall performance of the classifier is during several runs to the optimal solution, while the RMSE measures how concentrated the results of several runs are around the optimal solution. The STD measures how far the results of several runs are from the mean. Thus, in the current study, if the mean value is high and close to 100.00%, this means the classifier performance was good during the several runs, while a low value for RMSE and STD indicates that the classifier performed rather well during the many runs and frequently produced results similar to or almost equal to 100.00%. **Tables 6** and **7** present the statistical results for all experiments of the proposed FLN algorithm using WBCD and the WDBC database, respectively. In **Tables 6** and **7**, the best statistical results are presented in bold font. Equations 13–15 (54) are used to calculate the mean, RMSE, and STD.

**Table 6:** The statistical results for all experiments of the proposed FLN algorithm using the WBCD.

Results of the mean							
Hidden node number	Mean of accuracy	Mean of precision	Mean of recall	Mean of F-measure	Mean of G-mean	Mean of MCC	Mean of specificity
**25**	**98.37%**	**95.94%**	**99.40%**	**97.64%**	**97.65%**	**96.44%**	**97.85%**
50	98.10%	95.44%	99.11%	97.23%	97.26%	95.84%	97.59%
75	97.59%	94.25%	98.84%	96.47%	96.51%	94.73%	96.98%
100	96.88%	92.67%	98.36%	95.40%	95.46%	93.16%	96.17%
125	95.82%	89.97%	97.94%	93.75%	93.86%	90.84%	94.83%
150	94.92%	87.50%	97.82%	92.31%	92.49%	88.90%	93.64%
175	94.15%	85.69%	97.32%	91.07%	91.29%	87.20%	92.78%
200	91.98%	80.28%	96.26%	87.43%	87.85%	82.45%	90.28%
Results of the RMSE							
Hidden node number	RMSE of accuracy	RMSE of precision	RMSE of recall	RMSE ofF-measure	RMSE of G-mean	RMSE of MCC	RMSE of specificity
**25**	**1.7575**	**4.3390**	**1.0089**	**2.5554**	**2.5345**	**3.8464**	**2.2937**
50	2.0499	4.9457	1.2924	2.9908	2.9634	4.4871	2.6037
75	2.6224	6.3556	1.5575	3.8557	3.8106	5.7392	3.3211
100	3.3527	7.9543	2.0708	4.9616	4.8942	7.3411	4.1271
125	4.4468	10.6990	2.5004	6.6670	6.5500	9.7387	5.4868
150	5.3146	13.1820	2.7296	8.0834	7.8851	11.6033	6.6633
175	6.1308	15.0680	3.1271	9.4140	9.1632	13.4068	7.5565
200	8.3516	20.6529	4.2684	13.2125	12.7320	18.2805	10.0920
Results of the STD with N							
Hidden node number	STD of accuracy	STD of precision	STD of recall	STD of F-measure	STD of G-mean	STD of MCC	STD of specificity
**25**	**0.6589**	**1.5426**	**0.8106**	**0.9676**	**0.9578**	**1.4448**	**0.8014**
50	0.7635	1.9253	0.9403	1.1349	1.1187	1.6734	0.9905
75	1.0344	2.7076	1.0423	1.5525	1.5264	2.2686	1.3878
100	1.2224	3.0812	1.2603	1.8608	1.8184	2.6770	1.5469
125	1.5022	3.7298	1.4233	2.3304	2.2695	3.3025	1.8445
150	1.5521	4.1851	1.6419	2.5047	2.3931	3.3876	1.9891
175	1.8226	4.7324	1.6119	2.9893	2.8458	3.9887	2.2169
200	2.3316	6.1300	2.0616	4.0703	3.8005	5.1304	2.7240

Bold font refers to the highest achieved results.

**Table 7:** The statistical results for all experiments of the proposed FLN algorithm using the WDBC database.

Results of the mean							
Hidden node number	Mean of accuracy	Mean of precision	Mean of recall	Mean of F-measure	Mean of G-mean	Mean of MCC	Mean of specificity
**25**	**96.88%**	**94.84%**	**96.81%**	**95.80%**	**95.81%**	**93.35%**	**96.96%**
50	96.08%	93.38%	96.12%	94.70%	94.72%	91.65%	96.11%
75	95.71%	93.44%	95.11%	94.23%	94.25%	90.87%	96.13%
100	95.70%	93.53%	95.02%	94.23%	94.25%	90.86%	96.18%
125	95.25%	92.88%	94.45%	93.62%	93.64%	89.89%	95.79%
150	95.17%	92.69%	94.39%	93.50%	93.52%	89.71%	95.68%
175	94.88%	92.91%	93.51%	93.16%	93.19%	89.13%	95.79%
200	94.61%	92.47%	93.19%	92.78%	92.81%	88.54%	95.54%
Results of the RMSE							
Hidden node number	RMSE of accuracy	RMSE of precision	RMSE of recall	RMSE of F-measure	RMSE of G-mean	RMSE of MCC	RMSE of specificity
**25**	**3.3604**	**5.3628**	**4.0671**	**4.5016**	**4.4875**	**7.1472**	**3.1640**
50	4.1574	6.9386	4.7656	5.6179	5.5951	8.8628	4.0557
75	4.5501	6.9175	5.8042	6.0980	6.0753	9.6705	4.0678
100	4.5764	6.7640	6.0521	6.1079	6.0840	9.7061	3.9877
125	4.9787	7.4739	6.3851	6.6774	6.6559	10.5947	4.3954
150	5.0665	7.6674	6.3486	6.8084	6.7888	10.7952	4.5099
175	5.4621	7.5551	7.5229	7.2698	7.2450	11.5833	4.4696
200	5.6414	8.0191	7.5531	7.5441	7.5185	11.9856	4.7260
Results of the STD							
Hidden node number	STD of accuracy	STD of precision	STD of recall	STD of F-measure	STD of G-mean	STD of MCC	STD of specificity
**25**	**1.2411**	**1.4741**	**2.5196**	**1.6146**	**1.6111**	**2.6130**	**0.8608**
50	1.3900	2.0625	2.7622	1.8615	1.8585	2.9661	1.1555
75	1.5097	2.1875	3.1312	1.9762	1.9704	3.1816	1.2445
100	1.5550	1.9767	3.4446	2.0033	1.9987	3.2577	1.1302
125	1.4962	2.2569	3.1621	1.9647	1.9660	3.1766	1.2727
150	1.5286	2.3057	2.9746	2.0245	2.0227	3.2484	1.3086
175	1.8952	2.5998	3.8113	2.4612	2.4583	3.9931	1.4889
200	1.6594	2.7544	3.2715	2.1920	2.1848	3.5074	1.5550

Bold font refers to the highest achieved results.


(13) $$\mu =\;\frac{\sum_{i=1}^{N}X_{i}}{N}$$

(14) $$RMSE=\;\sqrt{\frac{\sum_{i=1}^{N}{\left(X_{i}-o\right)}^{2}}{N}}$$

(15) $$SDT=\;\sqrt{\frac{\sum_{i=1}^{N}{\left(X_{i}-\;\mu \right)}^{2}}{N}}$$

where μ refers to the mean of the population, *X_i_* represents each value of the population, *N* denotes the number of values in the population, and *o* refers to the observed/optimal value (i.e., 100.00%).

According to the results in **Tables 6** and **7**, the mean values of all measurements are close to 100.00%; that means the achieved accuracy, precision, recall, F-measure, G-mean, MCC, and specificity by the FLN algorithm were quite close to 100.00% most of the time during the 50 runs. While the values of both RMSE and STD are low (i.e., close to zero), which proves the effectiveness of the FLN algorithm in terms of achieving a high classification performance during the 50 runs. The proposed FLN algorithm has achieved the best statistical results when the number of the hidden neurons was 25 using both WBCD and the WDBC database.

For the WBCD (see **Table 6**):

- The mean value of the accuracy was 98.37%, precision was 95.94%, recall was 99.40%, F-measure was 97.64%, G-mean was 97.65%, MCC was 96.44%, and specificity was 97.85%.
- The RMSE values were accuracy 1.7575, precision 4.3390, recall 1.0089, F-measure 2.5554, G-mean 2.5345, MCC 3.8464, and specificity 2.2937.
- The STD values were accuracy 0.6589, precision 1.5426, recall 0.8106, F-measure 0.9676, G-mean 0.9578, MCC 1.4448, and specificity 0.8014.

For the WDBC database (see **Table 7**):

a) The mean values of the accuracy, precision, recall, F-measure, G-mean, MCC, and specificity were 96.88%, 94.84%, 96.81%, 95.80%, 95.81%, 93.35%, and 96.96%, respectively.
b) The RMSE value for the accuracy was 3.3604, precision 5.3628, recall 4.0671, F-measure 4.5016, G-mean 4.4875, MCC 7.1472, and specificity 3.1640.
c) The STD values for the accuracy was 1.2411, precision 1.4741, recall 2.5196, F-measure 1.6146, G-mean 1.6111, MCC 2.6130, and specificity 0.8608.

**Figure 4:**
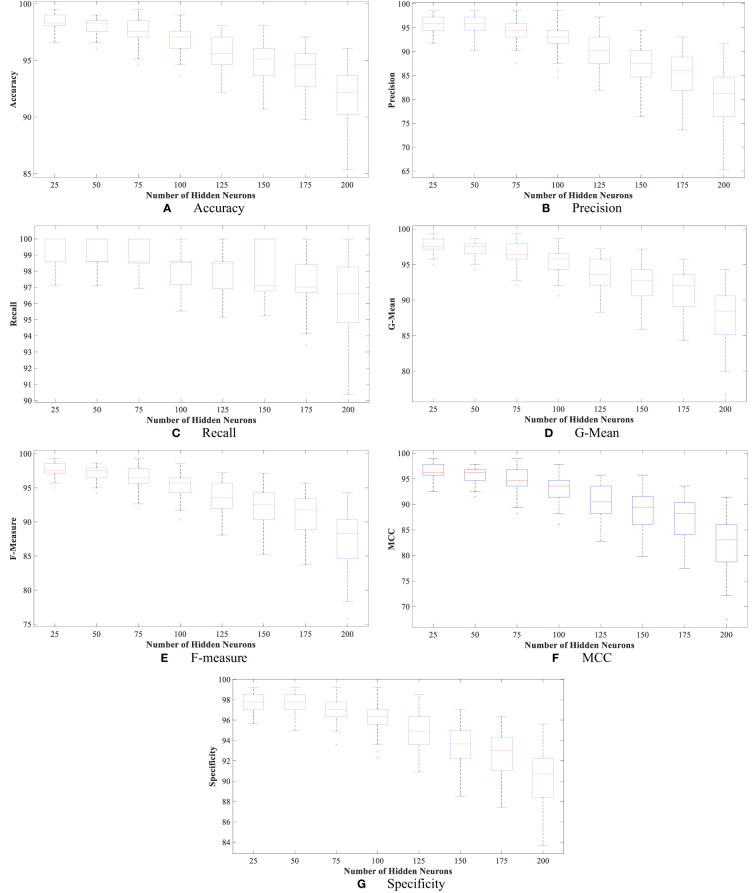
The proposed FLN algorithm’s results for the 50 runs in all experiments using WDBC database: **(A)** FLN algorithm accuracy results for the 50 runs in all experiments, **(B)** FLN algorithm precision results for the 50 runs in all experiments, **(C)** FLN algorithm recall results for the 50 runs in all experiments, **(D)** FLN algorithm G-mean results for the 50 runs in all experiments, **(E)** FLN algorithm F-measure results for the 50 runs in all experiments, **(F)** FLN algorithm MCC results for the 50 runs in all experiments, and **(G)** FLN algorithm specificity results for the 50 runs in all experiments.

**Figure 5:**
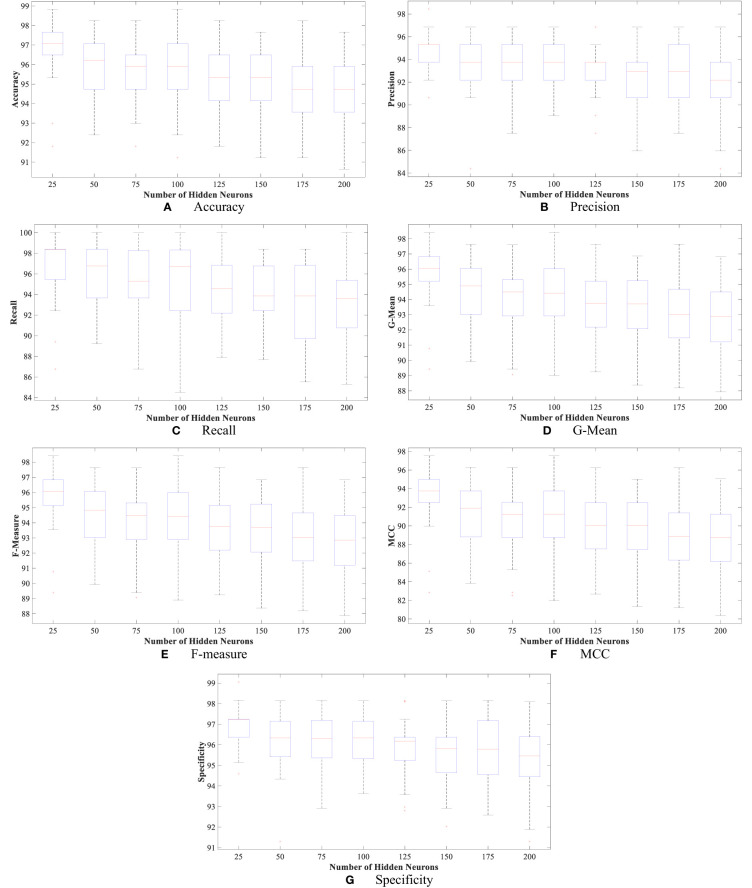
The proposed FLN algorithm’s results for the 50 runs in all experiments using the WBCD. **(A)** FLN algorithm accuracy results for the 50 runs in all experiments, **(B)** FLN algorithm precision results for the 50 runs in all experiments, **(C)** FLN algorithm recall results for the 50 runs in all experiments, **(D)** FLN algorithm G-mean results for the 50 runs in all experiments, **(E)** FLN algorithm F-measure results for the 50 runs in all experiments, **(F)** FLN algorithm MCC results for the 50 runs in all experiments, and **(G)** FLN algorithm specificity results for the 50 runs in all experiments.

Moreover, **Figures 4** and **5** show the boxplot of all evaluation measurements’ results during the 50 runs for the eight experiments using the WBCD and WDBC database.

**Figure 6:**
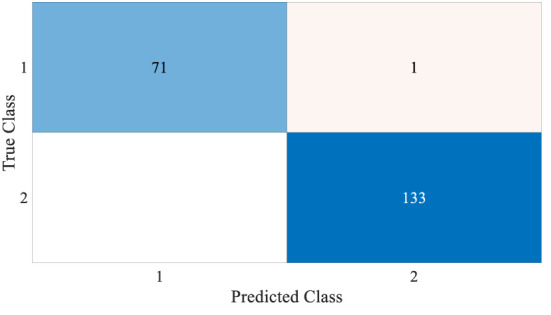
Confusion matrix for the best achieved results by the FLN algorithm using the WBCD.

**Figure 7:**
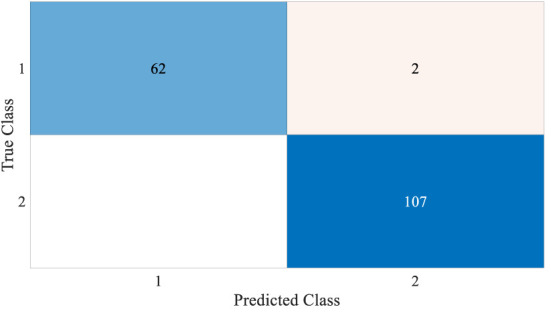
Confusion matrix for the best achieved results by the FLN algorithm using the WDBC database.

**Figures 4** and **5** show the results, which clearly show that the proposed FLN algorithm has done well across 50 runs in all trials utilizing the WBCD and WDBC database. For both the WBCD and WDBC datasets, the best 50 runs’ results of the proposed FLN algorithm were obtained using 25 hidden neurons (see **Figures 4**, **5**). For the WBCD, the range of accuracy is 96.59%–99.51%, precision 91.67%–98.61%, recall 97.14%–100.00%, F-measure 94.96%–99.30%, G-mean 95.03%–99.30%, MCC 92.52%–98.93%, and specificity 95.65%–99.25%, while for the WBCD, the respective ranges of accuracy, precision, recall, F-measure, G-mean, MCC, and specificity are 91.81%–98.83%, 90.63%–98.44%, 86.77%–100.00%, 89.39%–98.41%, 89.44%–98.43%, 82.84%–97.52%, and 94.60%–99%. The FLN algorithm’s best outcomes using the WBCD and WDBC database are shown in **Table 8**. Additionally, utilizing the WBCD and WDBC database, **Figures 6** and **7** display the confusion matrix for the FLN algorithm’s best outcomes, which is a clear indication that the proposed FLN algorithm on the WBCD was able to accurately classify 71 out of 72 malignant samples while successfully classifying all 33 testing samples of benign tissue (see **Figure 6**). For the WDBC database, it demonstrates that the suggested FLN method was capable of correctly classifying all of the testing samples, with the exception of two samples from the malignant category (64 malignant and 107 benign) (see **Figure 7**). The remarkable FLN performance is due to output layer neurons of the FLN that are also receiving external information directly through the input layer neurons, rather than just through the hidden layer neurons, which only receive the external information after it is modified.

**Table 8:** The highest achieved results of the FLN algorithm using the WBCD and WDBC database.

WBCD						
Accuracy	Precision	Recall	F-measure	G-mean	MCC	Specificity
99.51%	98.61%	100.00%	99.30%	99.30%	98.93%	99.25%
WDBC database						
Accuracy	Precision	Recall	F-measure	G-mean	MCC	Specificity
98.83%	98.44%	100.00%	98.41%	98.43%	97.52%	99.05%

**Figure 8:**
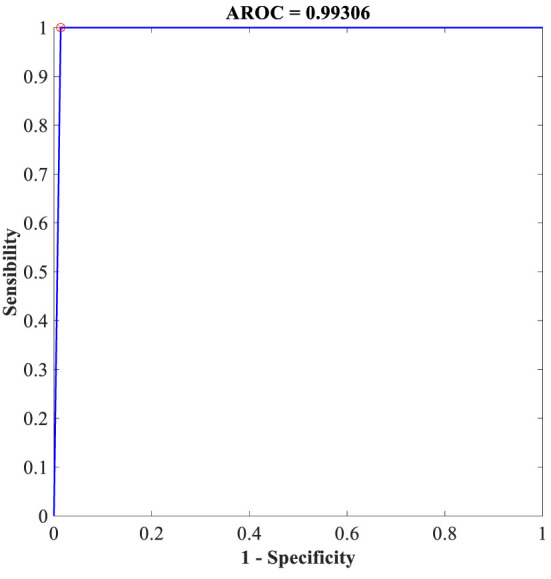
ROC for the best achieved results by the FLN algorithm using the WBCD.

**Figure 9:**
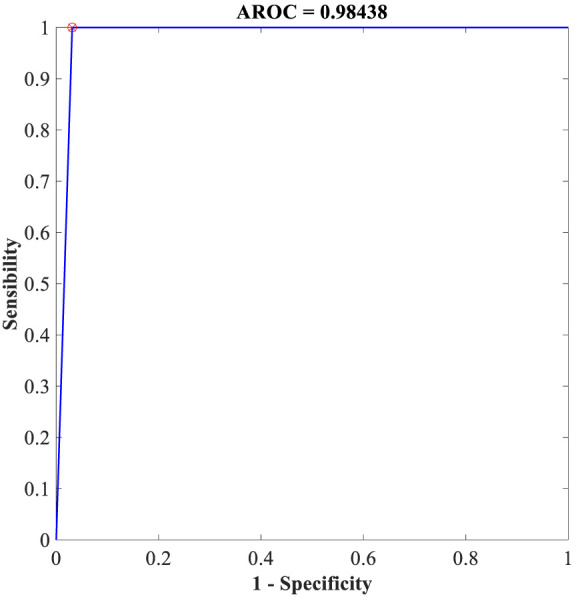
ROC for the best achieved results by the FLN algorithm using the WDBC database.

Additionally, the proposed FLN algorithm has been evaluated in terms of execution time and ROC. **Table 9** provides the execution time of the FLN algorithm based on the WBCD and WDBC database, where the results of the execution time in **Table 9** proves that the performance of the proposed FLN algorithm is quite fast and needs only a few milliseconds for the classification process, while **Figures 8** and **9** display the ROC of the best achieved results by the FLN algorithm using the WBCD and WDBC database. Based on the results in **Figures 8** and **9**, the proposed FLN algorithm achieved 0.99306 AROC using the WBCD and 0. 98438 AROC using the WDBC database. This obviously shows and proves that the proposed FLN algorithm correctly classified almost all the malignant and benign category points.

**Table 9:** The total run time for the 50 runs in all experiments of the proposed FLN algorithm using the WBCD and WDBC database.

WBCD	
Hidden node number	Execution time of the 50 runs
25	0.2766
50	0.3762
75	0.4507
100	0.5640
125	0.6485
150	0.7655
175	0.8224
200	0.9168
WDBC database	
Hidden node number	Execution time of the 50 runs
25	0.2437
50	0.3484
75	0.4276
100	0.5737
125	0.6042
150	0.7656
175	0.8242
200	0.9685

Additionally, the proposed FLN algorithm’s accuracy performance was compared with several recent studies (55–72) that made use of the same WBCD and WDBC datasets. Various ML and DM techniques have been reported in these publications for diagnosing the BC (i.e., diagnose whether the tumor is benign or malignant). **Table 10** provides a comparison of these studies’ rates of classification accuracy in BC diagnosis.

**Table 10:** The comparison of the accuracy rate (the best result) among the methods in diagnosing the BC based on the WBCD and WDBC database.

WBCD	
Method	Accuracy
Linear SVM (71)	96.72%
RF (66)	96.10%
XGBoost (72)	98.00%
XGBoost + RFE (56)	99.02%
KNN (57)	97.51%
C4.5 algorithm (69)	96.70%
MLP & LR (61)	98.00%
NB (59)	97.36%
ANN (64)	98.57%
**Proposed method (FLN)**	**99.51%**
WDBC database	
Method	Accuracy
Weighted vote-based ensemble (58)	95.09%
GA-classifier (55)	96.60%
EM-PCA-CART-fuzzy rule-based (67)	94.10%
LMNN-SRA (68)	96.66%
SVM with linear kernel (70)	98.24%
PCA + CNN (60)	96.40%
Bayesian network (65)	96.31%
Fuzzy-ID3+FUZZTDBD (63)	94.53%
HCRF (62)	97.05%
**Proposed method (FLN)**	**98.83%**

The results in **Table 10** demonstrate that the proposed FLN algorithm has outperformed all of its peers in terms of classification accuracy rate. However, there are still some challenges with the current work, which are as follows:

- The current work has considered the off-line classification task only while an online classification task is required.
- The present work has taken into account the task of breast cancer detection only and ignored the task of breast cancer stage classification.
- The fact that the FLN algorithm needs to be optimized in terms of the random generated input weights and biases has been ignored, where the random input weights and biases of the hidden layer are not the best parameters, which cannot always meet the training goals of the FLN to achieve the global minimum. In other words, based on given training data, there is no way to assure that the trained FLN is the most appropriate in performing the classification.

## 5 Conclusion

In this study, we have proposed a BC diagnosing classifier based on the FLN algorithm. The FLN algorithm was applied on two different BC databases WBCD and WDBC. Several experiments were implemented to assess the proposed FLN algorithm performance in diagnosing the BC by varying the hidden neuron number. The outcomes of each run were assessed based on accuracy, precision, recall, F-measure, G-mean, MCC, and specificity. The 50 runs’ results of each experiment were used to calculate the mean, RMSE, and STD in order to statistically assess the proposed FLN algorithm performance in diagnosing the BC. The statistical analysis proved the effectiveness of the proposed FLN algorithm in diagnosing the BC, and it confirms that the achieved results were not by accident. The performance of the FLN algorithm was impressive with an accuracy average reaching up to 98.37% using the WBCD and 96.88% using the WDBC database. The outstanding FLN performance is a result of the fact that external information is also directly received by FLN output layer neurons *via* input layer neurons, as opposed to merely through hidden layer neurons, which only receive external information after it has been transformed. Nevertheless, despite the fact that an online classification task was required, the current study only considered the offline classification problem. In addition, the present work has taken into account the task of breast cancer detection only and ignored the task of breast cancer stage classification. In addition, the need to optimize the FLN algorithm with respect to the input weights and biases generated at random has been omitted. The random input weights and biases of the hidden layer are not the best parameters, which cannot always meet the training goals of the FLN to achieve the global minimum. In other words, based on given training data, there is no way to assure that the trained FLN is the most appropriate in performing the classification. Therefore, in order to produce more appropriate biases and input weights for the FLN algorithm and reduce classification mistakes, the study’s future work should employ an optimization technique. Moreover, the proposed work was used to address the problems of the breast cancer stages classification as well as other healthcare applications.

## Data availability statement

Publicly available datasets were analyzed in this study. This data can be found at: WBCD: https://archive.ics.uci.edu/ml/datasets/Breast+Cancer+Wisconsin+%28Original%29; WDBC: https://archive.ics.uci.edu/ml/datasets/breast+cancer+wisconsin+(diagnostic).

## Author contributions

MAAA: conceptualization, methodology, writing of the original draft, software, writing of the review, and editing. MA: supervision, funding acquisition, and project administration. ST: supervision. FA-D: writing of the review and editing. AA: investigation. SK: investigation. All authors contributed to the article and approved the submitted version.
